# 1-Dichloro­acetyl-*r*-2,*c*-6-bis­(4-methoxy­phen­yl)-*t*-3,*t*-5-dimethyl­piperidin-4-one

**DOI:** 10.1107/S1600536809054725

**Published:** 2010-01-09

**Authors:** K. Ravichandran, P. Ramesh, C. Neeladevi, S. Ponnuswamy, M. N. Ponnuswamy

**Affiliations:** a Centre of Advanced Study in Crystallography and Biophysics, University of Madras, Guindy Campus, Chennai 600 025, India; b Department of Chemistry, Government Arts College (Autonomous), Coimbatore 641 018, India

## Abstract

In the title compound, C_23_H_25_Cl_2_NO_4_, the piperidine ring adopts a distorted boat conformation. The dihedral angle between the benzene rings is 54.8 (1)°. In the crystal, the mol­ecules are linked into a two-dimensional network parallel to the *ab* plane by C—H⋯O hydrogen bonds.

## Related literature

For the biological properties of piperidin-4-one compounds, see: El-Subbagh *et al.* (2000 ▶); Jerom & Spencer (1988 ▶); Perumal *et al.* (2001 ▶); Hagenbach & Gysin (1952 ▶); Mobio *et al.* (1989 ▶); Katritzky & Fan (1990 ▶); Ganellin & Spickett (1965 ▶). For ring puckering parameters, see: Cremer & Pople (1975 ▶). For asymmetry parameters, see: Nardelli (1983 ▶). For hydrogen-bond motifs, see: Bernstein *et al.* (1995 ▶).
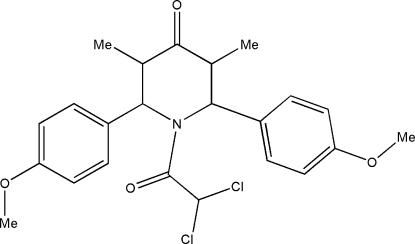


## Experimental

### 

#### Crystal data

C_23_H_25_Cl_2_NO_4_
*M* *_r_* = 450.34
Monoclinic, 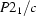

*a* = 8.1251 (7) Å
*b* = 9.9702 (9) Å
*c* = 27.649 (2) Å
β = 92.265 (5)°
*V* = 2238.0 (3) Å^3^
*Z* = 4
Mo *K*α radiation
μ = 0.32 mm^−1^
*T* = 293 K
0.27 × 0.26 × 0.23 mm

#### Data collection

Bruker SMART APEXII area-detector diffractometer
Absorption correction: multi-scan (*SADABS*; Bruker, 2008 ▶) *T*_min_ = 0.917, *T*_max_ = 0.929
19833 measured reflections
5379 independent reflections
3699 reflections with *I* > 2σ(*I*)
*R*_int_ = 0.026

#### Refinement

*R*[*F* ^2^ > 2σ(*F* ^2^)] = 0.046
*wR*(*F* ^2^) = 0.123
*S* = 1.03
5379 reflections
276 parameters
H-atom parameters constrained
Δρ_max_ = 0.27 e Å^−3^
Δρ_min_ = −0.36 e Å^−3^

### 

Data collection: *APEX2* (Bruker, 2008 ▶); cell refinement: *SAINT* (Bruker, 2008 ▶); data reduction: *SAINT*; program(s) used to solve structure: *SHELXS97* (Sheldrick, 2008 ▶); program(s) used to refine structure: *SHELXL97* (Sheldrick, 2008 ▶); molecular graphics: *ORTEP-3* (Farrugia, 1997 ▶); software used to prepare material for publication: *SHELXL97* and *PLATON* (Spek, 2009 ▶).

## Supplementary Material

Crystal structure: contains datablocks global, I. DOI: 10.1107/S1600536809054725/ci2992sup1.cif

Structure factors: contains datablocks I. DOI: 10.1107/S1600536809054725/ci2992Isup2.hkl

Additional supplementary materials:  crystallographic information; 3D view; checkCIF report

## Figures and Tables

**Table 1:** Hydrogen-bond geometry (Å, °)

*D*—H⋯*A*	*D*—H	H⋯*A*	*D*⋯*A*	*D*—H⋯*A*
C2—H2⋯O3^i^	0.98	2.40	3.326 (2)	158
C5—H5⋯O1^ii^	0.98	2.59	3.408 (2)	141

Symmetry codes: (i) 
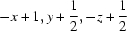
; (ii) 
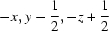
.

**Fig. 1.**
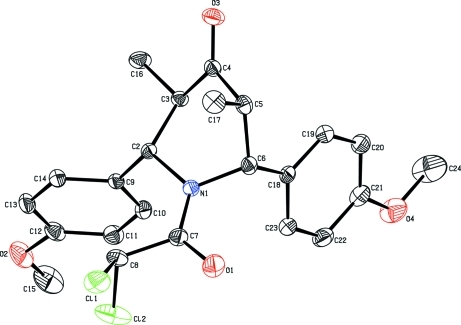
Molecular structure of the title compound. Displacement ellipsoids are drawn at the 30% probability level. H atoms have been omitted for clarity.

**Fig. 2.**
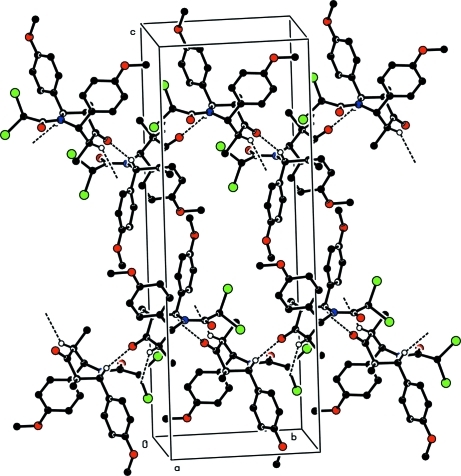
The crystal packing of the title compound, viewed along the a axis. H atoms not involved in hydrogen bonding have been omitted for clarity.

## supplementary crystallographic information

### Comment

Piperidin-4-one derivatives possess varied biological properties such as antiviral, antitumour (El-Subbagh *et al.*, 2000), analgesic (Jerom & Spencer, 1988), local anaesthetic (Perumal *et al.*, 2001; Hagenbach & Gysin, 1952), antimicrobial, bactericidal, fungicidal, herbicidal, insecticidal, antihistaminic, anti-inflammatory, anticancer, *CNS* stimulant and depressant activities (Mobio *et al.*, 1989; Katritzky & Fan, 1990; Ganellin & Spickett, 1965). In view of these importance and to ascertain the molecular conformation, a crystallographic study of the title compound has been carried out.

The *ORTEP* diagram of the title compound is shown in Fig.1. The piperidine ring adopts a distorted boat conformation with the puckering parameters (Cremer & Pople, 1975) and the asymmetry parameters (Nardelli, 1983) are: q_2_ = 0.737 (2) Å, q_3_ = 0.013 (2) Å, φ_2_ = 285.4 (1)° and Δ_s_(C3 or C6) = 19.0 (2)°. The sum of the bond angles around the atom N1 (359.1°) of the piperidine ring is in accordance with *sp*^2^ hybridization.

The crystal packing is stabilized by C—H···O intermolecular interactions. Atom C2 of the molecule at (*x*, *y*, *z*) donates a proton to atom O3 of the molecule at (1 - *x*, 1/2 + *y*, 1/2 - *z*), forming a C5 (Bernstein *et al.*, 1995) zigzag chain running along the *b* axis. The chains are cross linked *via* C5—H5···O1 intermolecular interactions, forming a two-dimensional network parallel to the *ab* plane.

### Experimental

To a solution of r-2,c-6-bis(4-methoxyphenyl)-t-3,t-5-dimethylpiperidin-4-one (1.69 g) in anhydrous benzene (60 ml) was added triethylamine (2.08 ml) and dichloroacetylchloride (1.42 ml). The reaction mixture was allowed to stir at room temperature for 8 hr and the solution was washed with water (4 × 25 ml). The organic layer was dried over anhydrous sodium sulfate, passed through a short column of silica, evaporated and crystallized from benzene-petroleum ether (60–80° C) (9:1 v/v).

### Refinement

H atoms were positioned geometrically (C-H = 0.93–0.98 Å) and allowed to ride on their parent atoms, with U_iso_(H) = 1.5*U*_eq_(C_methyl_) and 1.2 *U*_eq_(C).

### Crystal data

**Table:**

C_23_H_25_Cl_2_NO_4_	*F*(000) = 944
*M**_r_* = 450.34	*D*_x_ = 1.337 Mg m^−^^3^
Monoclinic, *P*2_1_/*c*	Mo *K*α radiation, λ = 0.71073 Å
Hall symbol: -P 2ybc	Cell parameters from 2335 reflections
*a* = 8.1251 (7) Å	θ = 1.5–28.4°
*b* = 9.9702 (9) Å	µ = 0.32 mm^−^^1^
*c* = 27.649 (2) Å	*T* = 293 K
β = 92.265 (5)°	Block, colourless
*V* = 2238.0 (3) Å^3^	0.27 × 0.26 × 0.23 mm
*Z* = 4	

### Data collection

**Table:**

Bruker SMART APEXII area-detector diffractometer	5379 independent reflections
Radiation source: fine-focus sealed tube	3699 reflections with *I* > 2σ(*I*)
graphite	*R*_int_ = 0.026
ω and φ scans	θ_max_ = 28.4°, θ_min_ = 1.5°
Absorption correction: multi-scan (*SADABS*; Bruker, 2008)	*h* = −8→10
*T*_min_ = 0.917, *T*_max_ = 0.929	*k* = −13→13
19833 measured reflections	*l* = −36→36

### Refinement

**Table:**

Refinement on *F*^2^	Secondary atom site location: difference Fourier map
Least-squares matrix: full	Hydrogen site location: inferred from neighbouring sites
*R*[*F*^2^ > 2σ(*F*^2^)] = 0.046	H-atom parameters constrained
*wR*(*F*^2^) = 0.123	*w* = 1/[σ^2^(*F*_o_^2^) + (0.0497*P*)^2^ + 0.6929*P*] where *P* = (*F*_o_^2^ + 2*F*_c_^2^)/3
*S* = 1.03	(Δ/σ)_max_ = 0.001
5379 reflections	Δρ_max_ = 0.27 e Å^−^^3^
276 parameters	Δρ_min_ = −0.36 e Å^−^^3^
0 restraints	Extinction correction: *SHELXL97* (Sheldrick, 2008), Fc^*^=kFc[1+0.001xFc^2^λ^3^/sin(2θ)]^-1/4^
Primary atom site location: structure-invariant direct methods	Extinction coefficient: 0.0028 (7)

### Special details

**Table:**

Geometry. All e.s.d.'s (except the e.s.d. in the dihedral angle between two l.s. planes) are estimated using the full covariance matrix. The cell e.s.d.'s are taken into account individually in the estimation of e.s.d.'s in distances, angles and torsion angles; correlations between e.s.d.'s in cell parameters are only used when they are defined by crystal symmetry. An approximate (isotropic) treatment of cell e.s.d.'s is used for estimating e.s.d.'s involving l.s. planes.
Refinement. Refinement of *F*^2^ against ALL reflections. The weighted *R*-factor *wR* and goodness of fit *S* are based on *F*^2^, conventional *R*-factors *R* are based on *F*, with *F* set to zero for negative *F*^2^. The threshold expression of *F*^2^ > σ(*F*^2^) is used only for calculating *R*-factors(gt) *etc*. and is not relevant to the choice of reflections for refinement. *R*-factors based on *F*^2^ are statistically about twice as large as those based on *F*, and *R*- factors based on ALL data will be even larger.

### Fractional atomic coordinates and isotropic or equivalent isotropic displacement parameters (Å^2^)

**Table:**

	*x*	*y*	*z*	*U*_iso_*/*U*_eq_	
Cl1	0.25916 (8)	1.01921 (6)	0.20873 (3)	0.0839 (2)	
Cl2	0.26208 (12)	0.94382 (8)	0.10863 (3)	0.1095 (3)	
O1	0.03043 (18)	0.80082 (15)	0.18222 (7)	0.0785 (5)	
O2	0.7057 (2)	0.78446 (18)	0.00094 (6)	0.0835 (5)	
O3	0.42430 (17)	0.31447 (14)	0.24506 (5)	0.0588 (4)	
O4	−0.1564 (2)	0.25273 (19)	0.03205 (6)	0.0880 (5)	
N1	0.23306 (16)	0.64601 (13)	0.17753 (5)	0.0373 (3)	
C2	0.41000 (18)	0.60777 (16)	0.17209 (6)	0.0347 (3)	
H2	0.4747	0.6462	0.1994	0.042*	
C3	0.42732 (19)	0.45310 (16)	0.17457 (6)	0.0375 (4)	
H3	0.3593	0.4165	0.1476	0.045*	
C4	0.3566 (2)	0.40055 (17)	0.22066 (6)	0.0400 (4)	
C5	0.1976 (2)	0.46357 (18)	0.23518 (6)	0.0429 (4)	
H5	0.1227	0.3916	0.2443	0.051*	
C6	0.1163 (2)	0.54088 (17)	0.19306 (6)	0.0407 (4)	
H6	0.0221	0.5882	0.2061	0.049*	
C7	0.1748 (2)	0.77330 (18)	0.17704 (7)	0.0466 (4)	
C8	0.2948 (2)	0.88677 (18)	0.16834 (7)	0.0536 (5)	
H8	0.4082	0.8544	0.1731	0.064*	
C9	0.48157 (19)	0.65819 (16)	0.12568 (6)	0.0367 (4)	
C10	0.4080 (2)	0.63098 (19)	0.08098 (6)	0.0451 (4)	
H10	0.3086	0.5846	0.0793	0.054*	
C11	0.4787 (2)	0.6711 (2)	0.03850 (7)	0.0530 (5)	
H11	0.4274	0.6514	0.0087	0.064*	
C12	0.6256 (3)	0.7404 (2)	0.04089 (8)	0.0563 (5)	
C13	0.7005 (2)	0.76910 (19)	0.08502 (8)	0.0561 (5)	
H13	0.7992	0.8165	0.0866	0.067*	
C14	0.6297 (2)	0.72769 (18)	0.12718 (7)	0.0471 (4)	
H14	0.6821	0.7467	0.1569	0.056*	
C15	0.6402 (4)	0.7474 (3)	−0.04496 (10)	0.1007 (10)	
H15A	0.6237	0.6521	−0.0460	0.151*	
H15B	0.7155	0.7728	−0.0693	0.151*	
H15C	0.5368	0.7921	−0.0510	0.151*	
C16	0.6029 (2)	0.4058 (2)	0.16746 (8)	0.0572 (5)	
H16A	0.6762	0.4496	0.1905	0.086*	
H16B	0.6341	0.4276	0.1353	0.086*	
H16C	0.6091	0.3105	0.1721	0.086*	
C17	0.2310 (3)	0.5525 (2)	0.27953 (7)	0.0608 (5)	
H17A	0.2733	0.4986	0.3060	0.091*	
H17B	0.1304	0.5947	0.2884	0.091*	
H17C	0.3103	0.6200	0.2721	0.091*	
C18	0.04865 (18)	0.45810 (17)	0.15056 (6)	0.0401 (4)	
C19	0.0299 (2)	0.32074 (18)	0.15241 (7)	0.0477 (4)	
H19	0.0635	0.2754	0.1805	0.057*	
C20	−0.0373 (2)	0.2483 (2)	0.11393 (7)	0.0546 (5)	
H20	−0.0482	0.1557	0.1162	0.066*	
C21	−0.0879 (2)	0.3135 (2)	0.07232 (7)	0.0572 (5)	
C22	−0.0711 (2)	0.4514 (2)	0.06939 (7)	0.0582 (5)	
H22	−0.1049	0.4963	0.0412	0.070*	
C23	−0.0045 (2)	0.5217 (2)	0.10811 (7)	0.0489 (4)	
H23	0.0053	0.6144	0.1058	0.059*	
C24	−0.1684 (5)	0.1120 (3)	0.03207 (13)	0.1282 (14)	
H24A	−0.0602	0.0738	0.0357	0.192*	
H24B	−0.2197	0.0825	0.0021	0.192*	
H24C	−0.2335	0.0838	0.0584	0.192*	

### Atomic displacement parameters (Å^2^)

**Table:**

	*U*^11^	*U*^22^	*U*^33^	*U*^12^	*U*^13^	*U*^23^
Cl1	0.0931 (5)	0.0454 (3)	0.1115 (5)	0.0192 (3)	−0.0188 (4)	−0.0273 (3)
Cl2	0.1680 (8)	0.0806 (5)	0.0815 (5)	0.0454 (5)	0.0254 (5)	0.0328 (4)
O1	0.0488 (8)	0.0526 (9)	0.1349 (15)	0.0178 (7)	0.0149 (9)	−0.0099 (9)
O2	0.0905 (12)	0.0833 (12)	0.0795 (11)	−0.0124 (10)	0.0404 (9)	0.0208 (9)
O3	0.0665 (9)	0.0521 (8)	0.0577 (8)	0.0080 (7)	0.0027 (7)	0.0194 (7)
O4	0.0982 (13)	0.0853 (12)	0.0777 (11)	0.0069 (10)	−0.0324 (9)	−0.0231 (9)
N1	0.0331 (7)	0.0329 (7)	0.0461 (7)	0.0036 (6)	0.0056 (6)	−0.0005 (6)
C2	0.0295 (8)	0.0346 (8)	0.0400 (8)	0.0026 (7)	0.0028 (6)	−0.0002 (7)
C3	0.0368 (8)	0.0347 (8)	0.0411 (8)	0.0054 (7)	0.0052 (6)	0.0028 (7)
C4	0.0446 (9)	0.0355 (9)	0.0399 (8)	−0.0032 (8)	−0.0004 (7)	0.0005 (7)
C5	0.0448 (9)	0.0443 (10)	0.0403 (9)	−0.0049 (8)	0.0108 (7)	0.0001 (8)
C6	0.0327 (8)	0.0413 (9)	0.0489 (9)	0.0009 (7)	0.0107 (7)	−0.0014 (8)
C7	0.0437 (10)	0.0386 (9)	0.0574 (10)	0.0089 (8)	0.0025 (8)	−0.0050 (8)
C8	0.0599 (12)	0.0335 (9)	0.0675 (12)	0.0111 (9)	0.0015 (9)	0.0006 (9)
C9	0.0332 (8)	0.0314 (8)	0.0457 (9)	0.0030 (7)	0.0047 (7)	0.0042 (7)
C10	0.0386 (9)	0.0488 (10)	0.0484 (9)	−0.0037 (8)	0.0052 (7)	0.0056 (8)
C11	0.0550 (11)	0.0589 (12)	0.0455 (10)	0.0019 (10)	0.0078 (8)	0.0089 (9)
C12	0.0575 (12)	0.0486 (11)	0.0645 (12)	0.0050 (10)	0.0234 (10)	0.0158 (10)
C13	0.0415 (10)	0.0453 (11)	0.0824 (14)	−0.0057 (9)	0.0143 (10)	0.0102 (10)
C14	0.0397 (9)	0.0403 (9)	0.0612 (11)	−0.0019 (8)	0.0021 (8)	0.0049 (8)
C15	0.132 (3)	0.105 (2)	0.0691 (17)	0.0001 (19)	0.0486 (17)	0.0209 (16)
C16	0.0496 (11)	0.0542 (11)	0.0691 (12)	0.0199 (9)	0.0174 (9)	0.0149 (10)
C17	0.0735 (14)	0.0637 (13)	0.0459 (10)	0.0017 (11)	0.0100 (9)	−0.0107 (9)
C18	0.0269 (8)	0.0437 (9)	0.0500 (9)	0.0019 (7)	0.0051 (7)	0.0009 (8)
C19	0.0434 (10)	0.0448 (10)	0.0549 (10)	0.0009 (8)	−0.0001 (8)	0.0033 (8)
C20	0.0490 (11)	0.0443 (10)	0.0701 (13)	0.0017 (9)	−0.0040 (9)	−0.0066 (10)
C21	0.0456 (11)	0.0642 (13)	0.0613 (12)	0.0067 (10)	−0.0060 (9)	−0.0129 (10)
C22	0.0495 (11)	0.0697 (14)	0.0545 (11)	0.0055 (10)	−0.0087 (9)	0.0068 (10)
C23	0.0390 (9)	0.0470 (10)	0.0606 (11)	0.0019 (8)	0.0002 (8)	0.0063 (9)
C24	0.163 (3)	0.085 (2)	0.131 (3)	0.030 (2)	−0.063 (2)	−0.055 (2)

### Geometric parameters (Å, °)

**Table:**

Cl1—C8	1.7609 (19)	C10—H10	0.93
Cl2—C8	1.757 (2)	C11—C12	1.379 (3)
O1—C7	1.218 (2)	C11—H11	0.93
O2—C12	1.376 (2)	C12—C13	1.372 (3)
O2—C15	1.406 (3)	C13—C14	1.383 (3)
O3—C4	1.210 (2)	C13—H13	0.93
O4—C21	1.367 (2)	C14—H14	0.93
O4—C24	1.406 (4)	C15—H15A	0.96
N1—C7	1.354 (2)	C15—H15B	0.96
N1—C6	1.489 (2)	C15—H15C	0.96
N1—C2	1.501 (2)	C16—H16A	0.96
C2—C9	1.516 (2)	C16—H16B	0.96
C2—C3	1.550 (2)	C16—H16C	0.96
C2—H2	0.98	C17—H17A	0.96
C3—C4	1.512 (2)	C17—H17B	0.96
C3—C16	1.523 (2)	C17—H17C	0.96
C3—H3	0.98	C18—C19	1.379 (2)
C4—C5	1.506 (2)	C18—C23	1.388 (2)
C5—C6	1.525 (2)	C19—C20	1.380 (3)
C5—C17	1.529 (2)	C19—H19	0.93
C5—H5	0.98	C20—C21	1.370 (3)
C6—C18	1.521 (2)	C20—H20	0.93
C6—H6	0.98	C21—C22	1.384 (3)
C7—O1	1.218 (2)	C22—C23	1.373 (3)
C7—C8	1.519 (3)	C22—H22	0.93
C8—H8	0.98	C23—H23	0.93
C9—C10	1.378 (2)	C24—H24A	0.96
C9—C14	1.388 (2)	C24—H24B	0.96
C10—C11	1.386 (2)	C24—H24C	0.96
			
C12—O2—C15	117.82 (19)	C13—C12—C11	119.99 (17)
C21—O4—C24	117.9 (2)	O2—C12—C11	123.9 (2)
C7—N1—C6	115.85 (14)	C12—C13—C14	120.19 (18)
C7—N1—C2	124.96 (14)	C12—C13—H13	119.9
C6—N1—C2	118.31 (12)	C14—C13—H13	119.9
N1—C2—C9	113.69 (12)	C13—C14—C9	120.84 (18)
N1—C2—C3	109.51 (12)	C13—C14—H14	119.6
C9—C2—C3	109.29 (13)	C9—C14—H14	119.6
N1—C2—H2	108.1	O2—C15—H15A	109.5
C9—C2—H2	108.1	O2—C15—H15B	109.5
C3—C2—H2	108.1	H15A—C15—H15B	109.5
C4—C3—C16	113.00 (14)	O2—C15—H15C	109.5
C4—C3—C2	110.20 (13)	H15A—C15—H15C	109.5
C16—C3—C2	112.72 (14)	H15B—C15—H15C	109.5
C4—C3—H3	106.8	C3—C16—H16A	109.5
C16—C3—H3	106.8	C3—C16—H16B	109.5
C2—C3—H3	106.8	H16A—C16—H16B	109.5
O3—C4—C5	121.55 (15)	C3—C16—H16C	109.5
O3—C4—C3	122.41 (16)	H16A—C16—H16C	109.5
C5—C4—C3	116.02 (14)	H16B—C16—H16C	109.5
C4—C5—C6	110.96 (13)	C5—C17—H17A	109.5
C4—C5—C17	109.19 (14)	C5—C17—H17B	109.5
C6—C5—C17	112.17 (15)	H17A—C17—H17B	109.5
C4—C5—H5	108.1	C5—C17—H17C	109.5
C6—C5—H5	108.1	H17A—C17—H17C	109.5
C17—C5—H5	108.1	H17B—C17—H17C	109.5
N1—C6—C18	111.99 (13)	C19—C18—C23	116.98 (17)
N1—C6—C5	108.31 (13)	C19—C18—C6	123.20 (16)
C18—C6—C5	116.62 (14)	C23—C18—C6	119.76 (16)
N1—C6—H6	106.4	C18—C19—C20	122.15 (18)
C18—C6—H6	106.4	C18—C19—H19	118.9
C5—C6—H6	106.4	C20—C19—H19	118.9
O1—C7—N1	123.20 (17)	C21—C20—C19	119.69 (19)
O1—C7—C8	118.56 (16)	C21—C20—H20	120.2
N1—C7—C8	118.22 (15)	C19—C20—H20	120.2
C7—C8—Cl2	108.39 (14)	O4—C21—C20	125.0 (2)
C7—C8—Cl1	109.58 (13)	O4—C21—C22	115.48 (19)
Cl2—C8—Cl1	109.31 (10)	C20—C21—C22	119.56 (19)
C7—C8—H8	109.8	C23—C22—C21	119.88 (19)
Cl2—C8—H8	109.8	C23—C22—H22	120.1
Cl1—C8—H8	109.8	C21—C22—H22	120.1
C10—C9—C14	118.03 (16)	C22—C23—C18	121.74 (18)
C10—C9—C2	121.78 (14)	C22—C23—H23	119.1
C14—C9—C2	120.10 (15)	C18—C23—H23	119.1
C9—C10—C11	121.56 (17)	O4—C24—H24A	109.5
C9—C10—H10	119.2	O4—C24—H24B	109.5
C11—C10—H10	119.2	H24A—C24—H24B	109.5
C12—C11—C10	119.40 (18)	O4—C24—H24C	109.5
C12—C11—H11	120.3	H24A—C24—H24C	109.5
C10—C11—H11	120.3	H24B—C24—H24C	109.5
C13—C12—O2	116.11 (19)		
			
C7—N1—C2—C9	−57.4 (2)	N1—C2—C9—C10	−54.2 (2)
C6—N1—C2—C9	133.76 (14)	C3—C2—C9—C10	68.44 (19)
C7—N1—C2—C3	179.99 (15)	N1—C2—C9—C14	129.30 (15)
C6—N1—C2—C3	11.19 (18)	C3—C2—C9—C14	−108.02 (17)
N1—C2—C3—C4	−54.86 (17)	C14—C9—C10—C11	0.1 (3)
C9—C2—C3—C4	180.00 (13)	C2—C9—C10—C11	−176.45 (16)
N1—C2—C3—C16	177.88 (14)	C9—C10—C11—C12	−0.3 (3)
C9—C2—C3—C16	52.74 (18)	C15—O2—C12—C13	174.8 (2)
C16—C3—C4—O3	−10.3 (2)	C15—O2—C12—C11	−5.1 (3)
C2—C3—C4—O3	−137.45 (17)	C10—C11—C12—C13	0.0 (3)
C16—C3—C4—C5	168.53 (15)	C10—C11—C12—O2	179.92 (19)
C2—C3—C4—C5	41.42 (19)	O2—C12—C13—C14	−179.40 (18)
O3—C4—C5—C6	−165.48 (16)	C11—C12—C13—C14	0.5 (3)
C3—C4—C5—C6	15.6 (2)	C12—C13—C14—C9	−0.8 (3)
O3—C4—C5—C17	70.4 (2)	C10—C9—C14—C13	0.5 (3)
C3—C4—C5—C17	−108.51 (17)	C2—C9—C14—C13	177.04 (16)
C7—N1—C6—C18	105.44 (17)	N1—C6—C18—C19	138.12 (16)
C2—N1—C6—C18	−84.76 (17)	C5—C6—C18—C19	12.6 (2)
C7—N1—C6—C5	−124.58 (15)	N1—C6—C18—C23	−44.8 (2)
C2—N1—C6—C5	45.23 (18)	C5—C6—C18—C23	−170.39 (14)
C4—C5—C6—N1	−58.81 (17)	C23—C18—C19—C20	0.5 (3)
C17—C5—C6—N1	63.61 (17)	C6—C18—C19—C20	177.62 (16)
C4—C5—C6—C18	68.56 (18)	C18—C19—C20—C21	−0.1 (3)
C17—C5—C6—C18	−169.01 (14)	C24—O4—C21—C20	−3.5 (4)
C6—N1—C7—O1	−9.9 (3)	C24—O4—C21—C22	176.7 (3)
C2—N1—C7—O1	−178.98 (17)	C19—C20—C21—O4	−179.80 (19)
C6—N1—C7—C8	171.65 (15)	C19—C20—C21—C22	−0.1 (3)
C2—N1—C7—C8	2.6 (2)	O4—C21—C22—C23	179.61 (18)
O1—C7—C8—Cl2	−74.4 (2)	C20—C21—C22—C23	−0.1 (3)
N1—C7—C8—Cl2	104.09 (17)	C21—C22—C23—C18	0.6 (3)
O1—C7—C8—Cl1	44.8 (2)	C19—C18—C23—C22	−0.7 (3)
N1—C7—C8—Cl1	−136.69 (15)	C6—C18—C23—C22	−177.94 (16)

### Hydrogen-bond geometry (Å, °)

**Table:**

*D*—H···*A*	*D*—H	H···*A*	*D*···*A*	*D*—H···*A*
C2—H2···O3^i^	0.98	2.40	3.326 (2)	158
C5—H5···O1^ii^	0.98	2.59	3.408 (2)	141

Symmetry codes: (i) −*x*+1, *y*+1/2, −*z*+1/2; (ii) −*x*, *y*−1/2, −*z*+1/2.
